# The psychometric evaluation of the Revised Exercise Addiction Inventory: Improved psychometric properties by changing item response rating

**DOI:** 10.1556/2006.8.2019.06

**Published:** 2019-03-28

**Authors:** Szabo Attila, Pinto Amit, Griffiths Mark D., Kovácsik Rita, Demetrovics Zsolt

**Affiliations:** 1 Institute of Health Promotion and Sport Sciences, Faculty of Education and Psychology, ELTE Eötvös Loránd University, Budapest, Hungary; 2 Institute of Psychology, ELTE Eötvös Loránd University, Budapest, Hungary; 3 Department of Psychology, The Nottingham Trent University, Nottingham, UK

**Keywords:** behavioral addiction, exercise addiction, exercise dependence, psychometric, questionnaires

## Abstract

**Background:**

The Exercise Addiction Inventory (EAI) is a short, valid, and reliable instrument used to assess the risk for exercise addiction, and has already been used in numerous published studies. The EAI contains six items, rated on a 5-point scale (*strongly agree* to *strongly disagree*), which are based on the components model of addiction. The middle of the original scale (scoring 3 out of 5) reflects neither agreement nor disagreement, which conveys neutrality. However, the present authors believe that individual who provides a neutral opinion on each item (i.e., scoring 3) is a conceptual dilemma because it artificially increases the total score obtainable on the scale without yielding agreement or disagreement with a particular item. Indeed, the six items of the EAI are phrased in such way that respondents can either agree or disagree in the slightly to strongly range.

**Methods:**

This study modified the EAI from a 5-point rating scale to a 6-point one, so that it eliminated a middle neutral response. A total of 277 exercising participants completed the Revised Exercise Addiction Inventory (EAI-R) and Exercise Dependence Scale.

**Results:**

All psychometric properties of the EAI-R were superior to the originally published scale.

**Conclusion:**

Considering these findings, it is recommended that scholars now use the EAI-R in the future research if they need to assess the risk of exercise addiction.

## Introduction

Scholars have asserted that exercise addiction is a psychological dysfunction in which the exerciser loses control over their exercise behaviour (Szabo, 2010). The affected individual behaves compulsively, exhibits withdrawal symptoms when exercise is not possible, and – due to extreme volumes of exercise – experiences conflict as well as negative life consequences (Szabo, Griffiths, & Demetrovics, 2016). In spite of increasing research attention, at present, there are no diagnosed cases of exercise addiction per se, because there are no official diagnostic criteria. While some authors classify problematic exercise as a behavioural addiction (Egorov & Szabo, 2013), exercise addiction is not included in the latest (fifth) edition of the “*Diagnostic and Statistical Manual of Mental Disorders*” (DSM-5) subsection of “Non-substance-related disorders” (American Psychiatric Association, 2013).

Researchers working in the area of exercise addiction have usually adapted the DSM criteria for substance dependence (Hausenblas & Downs, 2002), or used the components model of addiction (Griffiths, 2005) as the theoretical infrastructure for their work. The components model of addiction comprises six criteria, which are claimed to be present in all addictions irrespective of whether they are substance- or behavior-based addictions (Griffiths, 2005). The Exercise Addiction Inventory (EAI; Griffiths, Szabo, & Terry, 2005; Terry, Szabo, & Griffiths, 2004) is a popular tool employed in assessing the *risk* of exercise addiction, and was developed using the components model of addiction as its theoretical base. There are numerous (close to 100 to the best knowledge of these authors) published studies that used the instrument in the assessment of the risk of exercise addiction. It has been translated into several different languages (e.g., Chinese, Danish, German, French, Hungarian, Italian, and Spanish).

A conceptual problem with the original EAI, overlooked by the original developers and others, is that the rating on its Likert scale is not incremental (like a frequency scale from *never* to *always*), but ranges from “*strongly disagree*” to “*strongly agree*,” with a midpoint response “*neither agree nor disagree,*” which gives a score of 3 (out of 5). Therefore, the neutral answer can artificially increase the total score of the EAI. There are two possible solutions that may help in overcoming this issue. One is the use of a frequency scale on which the six symptoms of the components model (salience, conflict, withdrawal, mood modification, tolerance, and relapse; Griffiths, 2005) are rated on a frequency scale instead of the agree–disagree scale. In this case, the 5-point scale could be changed to: 1 = *never,* 2 = *sometimes,* 3 = *often,* 4 = *very often,* and 5 = *always*. Here, the midpoint of the scale adds a conceptually sound increment to the total rating. However, the “*sometimes*,” “*often*,” and “*very often*” are more arbitrary than “*yes*” or “*no*” answers (Brown, 2004) mirrored by agreement or disagreement with a given item/symptom. Therefore, an agree–disagree scale seems to be more appropriate, but the elimination of the middle neutral response is necessary. By increasing the Likert scale to six points (Brown, 2004), three “agree” and three “disagree” responses can be obtained: 1 = *strongly disagree,* 2 = *disagree,* 3 = *slightly disagree,* 4 = *slightly agree,* 5 = *agree,* and 6 = *strongly agree.* This spectrum facilitates the interpretation concerning the presence of the six symptoms in the components model of addiction by having three endorsing (agree) and three non-endorsing (disagree) responses that could be collapsed into two (yes/no) categories if necessary (Allen & Seaman, 2007). Indeed, a 6/6 or even a 5/6 agreement with the presence of the symptoms is likely to be a more accurate estimate of the risk of exercise addiction than a score of ≥24 on the original scale (Terry et al., 2004), which could comprise three agree (i.e., 3 × 5 = 15) and three neutral (3 × 3 = 9; 15 + 9 = 24) answers.

The aim of this study was therefore to revise the original EAI (Terry et al., 2004) by eliminating the midpoint neutral answer “*neither agree nor disagree*” and using a 6-point rating scale (Brown, 2004) while transforming it into a forced-choice tool with three levels of agreement and disagreement. A parallel objective of the study was to evaluate the psychometric properties of the Revised Exercise Addiction Inventory (EAI-R).

## Methods

### Participants

Participants were recruited via a call for participants posted on various social media and utilizing the snowball method (Goodman, 1961). The final sample comprised 277 participants in which most of them were men (*n* = 243). Their age ranged between 22 and 45 years (*M* = 30.47 ± *SD* = 4.97). All participants consented to participation in the study and confirmed that they exercised regularly at least three times per week for at least 30 min each time. An estimate of the total weekly volume of exercise was obtained by multiplying the frequency with the duration of exercise showing that participants exercised for an average of 254.50 (±*SD* = 193.18) min every week.

### Materials

#### Exercise Dependence Scale – Revised (EDS-R; Downs, Hausenblas, & Nigg, 2004)

The EDS-R is a 21-item instrument, which was originally based on the DSM-IV criteria for substance dependence. The responses are given on a 6-point Likert scale, ranging from 1 (*never*) to 6 (*always*). The ratings provide a total score for exercise dependence, which comprises the sum of ratings of seven components. The originally reported internal consistencies (Cronbach’s *α*) ranged between .78 and .95. In this study, the overall score of the EDS-R was used to assess the congruent validity of the EAI-R (see below). The internal reliability of the EDS-R in the present sample was .98.

#### Revised Exercise Addiction Inventory (EAI-R)

The original EAI (Griffiths et al., 2005; Terry et al., 2004) is based on Griffiths’ (2005) addiction components model and it was designed to assess six common symptoms of addiction. In the original EAI (Terry et al., 2004), the six items were rated on a 5-point Likert scale. In this study, a 6-point scale was used that yielded three *agree* and three *disagree* answers at three different levels (1 = *strongly disagree*, 2 = *disagree*, 3 = *slightly disagree*, 4 = *slightly agree*, 5 = *agree*, and 6 = *strongly agree*). The idea behind this change (as argued for in “Introduction” section) was to eliminate the midpoint uncertainty from the original scale. The psychometric properties of the revised tool are presented in “Results” section. The revised version of the EAI can be found in Appendix.

### Procedure

The participants completed the study anonymously using the *Qualtrics* online research platform (Qualtrics, 2017) having a unique uniform resource locator for the research. To access the questions and tools described in “Materials” section, participants had to read a consent form and agree to participate by selecting the “*I agree*” button. Only those with fully completed (100%) responses were included in this study. The data were downloaded in a Statistical Package for Social Sciences (version 24.0, Released 2016, IBM SPSS Statistics for Windows, IBM Corp., Armonk, NY) data file and analyzed with the same statistical software.

### Ethics

This study was conducted with permission obtained from the Research Ethics Committee acting in the Faculty of Education and Psychology at ELTE Eötvös Loránd University in Budapest. The study was totally anonymous, so there was no possibility to identify the respondents.

## Results

A principal components analysis confirmed, through both eigenvalue (4.087) and the scree-plot, that the six EAI-R items represent a single component explaining 68.12% of the variance. The modified scale’s concurrent validity with EDS-R was very good (*r* = .87). The EAI-R’s content validity was accepted on the basis of the original EAI scale (Terry et al., 2004). Construct validity was determined employing a cross-sectional one-way analysis of variance (ANOVA) for unequal sample sizes. This ANOVA was performed to determine if the scale could distinguish between higher and lower volumes of exercise. The ANOVA showed that participants who exercised more than 180 min every week (*n* = 108) scored significantly higher on the EAI-R (*M* = 25.64 ± *SD* = 5.22) than those who exercised less than 180 min per weak [*n* = 118, *M* = 13.17 ± *SD* =6.76, *F*(1, 224) = 243.50, *p* < .005, effect size (Cohen’s *d*) = 2.06]. The EAI-R shared a significant proportion of the variance with the weekly frequency of exercise (*r* = .612, *r*^2^ = .38%), and even more with the weekly exercise volume (*r* = .861, *r*^2^ = .74%). A second ANOVA testing for possible gender differences in EAI-R scores, in spite of large group-size differences, yielded no significant difference between the two genders, and that was also confirmed using a non-parametric Mann–Whitney *U* test, which is less sensitive to large sample size differences (Pett, 2015) and it is more appropriate for testing Likert-scale data in contrast with parametric tests (Allen & Seaman, 2007).

A confirmatory factor analysis was performed with the six items of the EAI-R in habitual exercisers and examined the one-factor solution. The fit indices indicated good fit [*χ*^2^ = 20.87, *df* = 9, *p* < .013, CMIN/DF = 2.319, GFI = 0.977, AGFI = 0.946, CFI = 0.988, TLI = 0.946, RMSEA = 0.069 (0.030–0.108), PCLOSE = 0.182, SRMR = 0.024]. Factor loadings were relatively high ranging from 0.635 to 0.870. This one-factor solution confirmed the theoretically proposed structure for the EAI-R. Internal consistency of the modified scale was very high (Cronbach α = .90).

### Prevalence of the risk of exercise addiction

The prevalence of the risk of exercise addiction was assessed based on the high scores. For the original EAI, it was suggested that a score ≥80% of the maximum score of 30 (i.e., ≥24) should reflect a risk score for exercise addiction. With an increase in the maximum value of the scale scores from 30 to 36, the 80% cut-off score was calculated to be 28.8 (29 to the nearest integer). Therefore, if the scores’ interpretation is based on the original scale (Terry et al., 2004), a score ≥29 represents a risk for exercise addiction. Consequently, in the present sample, 32/277 participants (11.5%) were classified to be at risk of exercise addiction based on the EAI-R, which was not statistically significantly different from the rate obtained using the EDS-R (25/277, 9.0%) as calculated with a McNemar’s test (*p* = .118, two-sided, exact test). The two scales agreed in 243 cases (out of 277) in the classifications (based on EDS-R and EAI-R scores) of “at risk,” “symptomatic,” and “asymptomatic” categories.

## Discussion

A principal component analysis confirmed that the six EAI-R items represented a single component explaining 68.12% of the variance, which was larger than the value reported in the original psychometric evaluation of the scale (55.9%; Griffiths et al., 2005). The modification of the EAI-R’s response rating scale resulted in higher internal consistency (Cronbach’s α) of .90 than that reported in the original scale (.84; Terry et al., 2004). The modified scale’s concurrent validity with EDS-R was also greater (*r* = .87) in this study than that reported for the original scale (*r* = .81; Griffiths et al., 2005). The EAI-R’s shared variance with the weekly frequency of exercise was also higher (*r*^2^ = .38%) than in the original study (*r*^2^ = .29). The EAI-R’s shared variance with the weekly volume of exercise (Frequency × Duration) was also very high.

While confirmatory factor analysis was not reported for the original inventory, in a cross-cultural evaluation of the EAI (Griffiths et al., 2015), this test was performed separately for data stemming from five nations. The model fit obtained in the present work was at least as good as those reported for several nations by Griffiths et al. (2015). Furthermore, in accordance with the originally reported results for the EAI (Terry et al., 2004), no gender differences were found in this study.

The prevalence of exercise addiction, using a cut-off value based on the original EAI, was similar to that obtained with the EDS-R, both being close to 10% in the current sample. However, the value estimated with the EAI-R (11.5%) was slightly higher than the value estimated with the EDS-R (9.0%). Nevertheless, using the classification proposed by Terry et al. (2004), the estimated prevalence of the risk of exercise addiction obtained with the EAI-R and EDS-R was not statistically significantly different from each other. In an earlier population-wide study, the EAI also yielded a greater risk of exercise addiction in regular exercisers than the EDS-R (Mónok et al., 2012). For example, the prevalence of the risk of exercise addiction in the general population was 0.3% based on the EDS and 0.5% based on the EAI. The respective values were 1.9% (EDS) and 3.2% (EAI) among the exercising population. Non-representative population studies have reported rates up to more than 10 times higher than these figures (Szabo, Griffiths, de la Vega Marcos, Mervó, & Demetrovics, 2015) but these findings should be treated cautiously, given the convenience sampling methods used. In this context, it should be noted that high EAI-R scores merely reflect the *possible* risk of exercise addiction and should not be interpreted as having diagnostic value, which could only be obtained via follow-up interviews with clinicians (Szabo et al., 2015). Furthermore, considering their theoretical foundations, the two scales measure similar but not totally identical psychological constructs and the EDS-R is rated on a frequency scale, whereas the EAI-R is rated on an agreement–disagreement scale. It was argued that the latter is more optimal than the former (Brown, 2004).

Due to several limitations, the present findings should be viewed as highly promising, but perhaps tentative. One limitation is the lack of examination of the test–retest reliability of the EAI-R, which was not possible due to the anonymous one-time only online data collection. Future studies should test the test–retest reliability of the EAI-R using a repeated measures research design. Although apparently making no difference, the large difference between men and women participants may be considered as another limitation of the study. Gender differences in exercise addiction have been reported in the literature, but the conclusion of a recent systematic review was that more research is needed in this area (Dumitru, Dumitru, & Maher, 2018). Indeed, such differences may have emerged if there were a larger representation of females in the total sample, which is a hypothesis that should be examined in future research. In this context, the age and type of exercise of the participants also merits specific scrutiny in future studies. Finally, all the data were self-reported and are therefore subject to well-known response biases.

## Conclusions

This study provides evidence for very good psychometric properties and good model fit with the theoretical structure of the EAI-R based on the components model of addiction (Griffiths, 2005). Despite the fact that this study simply modified the range of rating of the original EAI without any other changes, further confirmation of the current findings may be useful with a larger and more varied sample in future studies, especially the inclusion of more female participants. The test–retest reliability of the EAI-R should also be examined in a repeated measures research design. In the interim, based on the promising results presented, it is recommended that scholars start using the EAI-R instead of the original EAI, especially because the original scale had an artificial increment to the estimation of the risk of exercise addiction (because of its neutral midpoint score), which is no longer possible with the EAI-R.

## Appendix: The revised Exercise Addiction Inventory (EAI-R)

**Table:**

	Strongly disagree (1)	Disagree (2)	Slightly disagree (3)	Slightly agree (4)	Agree (5)	Strongly agree (6)
(1) Exercise is the most important thing in my life	○	○	○	○	○	○
(2) Conflicts have arisen between me and my family and/or my partner about the amount of exercise I do	○	○	○	○	○	○
(3) I use exercise as a way of changing my mood (e.g., to get a buzz, to escape, etc.)	○	○	○	○	○	○
(4) Over time I have increased the amount of exercise I do in a day	○	○	○	○	○	○
(5) If I have to miss an exercise session, I feel moody and irritable	○	○	○	○	○	○
(6) If I cut down the amount of exercise I do and then start again, I always end up exercising as often as I did before	○	○	○	○	○	○
